# The most commonly used disease severity scores are inappropriate for risk stratification of older emergency department sepsis patients: an observational multi-centre study

**DOI:** 10.1186/s13049-017-0436-3

**Published:** 2017-09-11

**Authors:** Bas de Groot, Frank Stolwijk, Mats Warmerdam, Jacinta A. Lucke, Gurpreet K. Singh, Mo Abbas, Simon P. Mooijaart, Annemieke Ansems, Laura Esteve Cuevas, Douwe Rijpsma

**Affiliations:** 1 Department of emergency medicine, Leiden University Medical Centre, Albinusdreef 2, 2300 RC Leiden, the Netherlands; 2 Department of Gerontology and Geriatrics, Leiden University Medical Centre, Albinusdreef 2, 2300 RC Leiden, The Netherlands; 3 Institute for Evidence-based Medicine in Old Age | IEMO, Albinusdreef 2, 2300 RC Leiden, The Netherlands; 4 Department of emergency medicine, Albert Schweitzer Ziekenhuis, Albert Schweitzerplaats 25, 3318 AT Dordrecht, the Netherlands; 5 Department of emergency medicine, Rijnstate Ziekenhuis, Wagnerlaan 55, 6815 AD Arnhem, the Netherlands

**Keywords:** Sepsis, Infectious diseases, Older patients, Emergency medical services, Risk stratification, Disease severity scores, qSOFA, Mortality

## Abstract

**Background:**

Sepsis recognition in older emergency department (ED) patients is difficult due to atypical symptom presentation. We therefore investigated whether the prognostic and discriminative performance of the five most commonly used disease severity scores were appropriate for risk stratification of older ED sepsis patients (≥70 years) compared to a younger control group (<70 years).

**Methods:**

This was an observational multi-centre study using an existing database in which ED patients who were hospitalized with a suspected infection were prospectively included. Patients were stratified by age < 70 and ≥70 years. We assessed the association with in-hospital mortality (primary outcome) and the area under the curve (AUC) with receiver operator characteristics of the Predisposition, Infection, Response, Organ dysfunction (PIRO), quick Sequential Organ Failure Assessment (qSOFA), Mortality in ED Sepsis (MEDS), and the Modified and National Early Warning (MEWS and NEWS) scores.

**Results:**

In-hospital mortality was 9.5% ((95%-CI); 7.4–11.5) in the 783 included older patients, and 4.6% (3.6–5.7) in the 1497 included younger patients. In contrast to younger patients, disease severity scores in older patients associated poorly with mortality. The AUCs of all disease severity scores were poor and ranged from 0.56 to 0.64 in older patients, significantly lower than the good AUC range from 0.72 to 0.86 in younger patients. The MEDS had the best AUC (0.64 (0.57–0.71)) in older patients. In older and younger patients, the newly proposed qSOFA score (Sepsis 3.0) had a lower AUC than the PIRO score (sepsis 2.0).

**Conclusion:**

The prognostic and discriminative performance of the five most commonly used disease severity scores was poor and less useful for risk stratification of older ED sepsis patients.

**Electronic supplementary material:**

The online version of this article (10.1186/s13049-017-0436-3) contains supplementary material, which is available to authorized users.

## Background

Sepsis recognition in older emergency department (ED) patients is difficult due to atypical symptom presentation. Delayed recognition of sepsis can result in delayed or even absent initiation of adequate treatment which has been shown to increase mortality and health-care costs [1–4]. Several disease severity scores have been specifically developed for ED patients who are hospitalized with a suspected infection and are supposed to help in sepsis recognition and risk stratification [5–13]. It is unclear however whether these disease severity scores are appropriate for risk stratification of older patients, i.e. have a high enough discriminative performance to identify high and low risk older patients which is needed for adequate disposition to a ward or intensive care unit (ICU). For example, it has been shown that in older patients vital signs detect cardiac arrest less accurately compared with non-elderly patients, which has important implications for how they are used for identifying critically ill patients. Churpek et al. suggested that more accurate methods for risk stratification of older patients are necessary with regard to the early detection cardiac arrest [14]. It is therefore possible that the currently available disease severity scores which are used for risk stratification of ED patients with a suspected infection may also be inappropriate for older patients due to the often absent classical symptoms such as fever, tachycardia and hypoxemia. Because these symptoms are an integral part of all the regularly used disease severity scores, their sensitivity will decrease in older patients.

If the prognostic and discriminative performance of the currently available disease severity scores are inappropriate for risk stratification of older ED patients at risk for sepsis, we need to develop a risk stratification tool specifically for older patients. This is especially important for the ED because delayed sepsis recognition could result in an inappropriate disposition to a ward rather than an ICU, increasing hospital length of stay and mortality [15]. In addition, in older patients in whom an ICU admission is not deemed desirable anymore, adequate initial fluid resuscitation should preferably take place in a monitored place like the ED because treatment monitoring is more difficult on a normal ward.

The aim of this study was therefore to assess if the prognostic and discriminative performance of the five most commonly used disease severity scores were appropriate for risk stratification of older ED sepsis patients (≥70 years) compared to a younger control group (<70 years).

## Methods

### Study design and setting

This was an observational study using an existing database in which ED patients were and are still prospectively collected as part of an ongoing quality improvement program in 3 Dutch EDs: Leiden University Medical Centre (LUMC; tertiary care centre with ~30,000 visits/year), Rijnstate Hospital (RH; urban care centre with ~30,000 visits/year), and the Albert Schweitzer Hospital (ASZ; urban care centre with ~25,000 visits/year). Patients were included from April 1st 2011 to February 1st 2016 in the LUMC, from March 1st 2012 to November 1st 2012 in the RH, and from September 1st 2015 to November 1st 2015 in the ASZ. After inclusion in the database, patients were stratified by age into an older (≥70 years) and younger (<70 years) group, as this is the cut-off which is also used in all Dutch government instated interventions for older people [16].

The study was approved by the medical ethics committee of the LUMC, who waived the need for individual informed consent as this was a pure observational study embedded in routine clinical care.

### Selection of participants

All consecutive ED patients of 17 years and older with a suspected infection and Manchester triage category yellow, orange or red [17] who received intravenous antibiotics in the ED and were subsequently admitted to the hospital were included in the database. Triage categories blue and green were excluded in the quality improvement program because most of these patients were expected to be at very low risk for mortality or admission. Patients who appeared to have no infection according to the final hospital discharge letter were excluded.

### Data collection

In all participating hospitals, the same “Surviving Sepsis Campaign-based” quality improvement program was used, in which a standard screening procedure was followed to optimize sepsis recognition, early ED resuscitation and disposition to an appropriate level of care. The quality improvement program is illustrated in Additional file 1 and has been described in detail elsewhere [18, 19].

Demographic and co-morbidity data, relevant time points and dates, laboratory variables, triage categories and vital signs, time to antibiotics, type of antibiotics, amount and type of fluids (L), administered oxygen (L/min), disposition and outcome variables were prospectively registered in the digital hospital information system Chipsoft Ezis (Chipsoft, Amsterdam, Netherlands) of each participating hospital. A medical student or registrar in emergency medicine subsequently transferred data from the electronic hospital information system to a web-based data collection file (PromiseBasic, Leiden, Netherlands, https://www.msbi.nl/promise/promise.aspx), which automatically calculated the Predisposition, Infection, Response and Organ failure (PIRO) score and the Mortality in ED Sepsis (MEDS) score. After the inclusion period, data of the three participating hospitals were transferred to one SPSS file (SPSS version 23.0, IBM, New York, USA).

Time to antibiotics was measured by subtraction of registration time at the ED desk from the registered time of antibiotic administration by the nurse. Time is zero was taken as the time at ED registration. The appropriateness of the initial dose of antibiotics administered in the ED was assessed in retrospect and is summarized in Additional file 2.

By means of an automated query in the digital hospital information system *all* ED patients who had been admitted with intravenous antibiotics were selected. Of these ED patients, we retrospectively investigated how many had been triaged as non-urgent but had been admitted with intravenous antibiotics. In this way, we could quantify the number of patients who had been missed by the screening procedure of the quality improvement program (which excluded non-urgent triage categories) because of atypical symptom presentation, which was expected to occur more often in older patients.

#### Disease severity scores

MEDS and PIRO scores are both a combination of age, comorbidities (predisposition factors) and acute physiology variables [7, 9]. The quick Sequential Organ Failure Assessment (qSOFA) score is a newly developed score that screens for low blood pressure (SBP ≤ 100 mmHg), high respiratory rate (≥22 per min), and altered mental status (Glasgow coma scale < 15) [12, 13]. The Modified Early Warning score (MEWS) incorporates temperature and urine production into the more common variables heart rate, systolic blood pressure, respiratory rate and altered mental status [10]. The National Early Warning Score (NEWS) does not use urine production, but instead incorporates arterial oxygen saturation and the use of supplemental oxygen [11]. The five scores have been originally developed for slightly different purposes. The MEDS and PIRO scores have been developed to predict in-hospital mortality in ED patients with a suspected infection and qSOFA and MEWS and NEWS to predict sepsis or clinical deterioration. However, this does not complicate the comparison of the prognostic and discriminative performance of these scores between older and younger patients.

All disease severity scores were calculated retrospectively so the treating physicians were not aware of the score at the time of ED presentation. Missing values were counted as normal, similar as in the APACHE score [20]. A patient was considered to have a “Do not resuscitate” (DNR) status if existing medical files already stated that the patient had a DNR code or when it was decided at the time of ED presentation or during hospital admission.

### Outcome measures

The primary outcome measure was in-hospital mortality.

Secondary outcome measures were ICU or MCU admission, an unanticipated transfer to an ICU or MCU within 48 h after being admitted to a ward [19], and the composite outcome of in-hospital mortality, ICU or MCU admission, or unanticipated transfer to an ICU or MCU within 48 h.

### Data analysis

#### Descriptives

Data are displayed as percentages, means and standard deviation for normally distributed variables or as median with interquartile range for non-normally distributed variables. Independent T-tests were used to assess differences between groups when normally distributed and with Mann-Whitney-U test for non-normally distributed variables. Chi-square test was used for categorical variables.

Each disease severity score was divided into 4 categories to allow comparison among the 5 individual scores: low (PIRO 0–6, qSOFA 0, MEDS 0–5, MEWS 0–3 and NEWS 0–3), moderate (PIRO 7–12, qSOFA 1, MEDS 6–9, MEWS 4–6 and NEWS 4–7), high (PIRO 13–18, qSOFA 2, MEDS 10–15, MEWS 7–9 and NEWS 8–11) and severe (PIRO ≥19, qSOFA 3, MEDS ≥16, MEWS ≥10 and NEWS ≥12). These values were chosen taking into account the individual score guidelines to best represent comparable disease severity categories.

#### Main analysis

The prognostic performance of all disease severity scores in both age groups was assessed by associating the aforementioned disease severity categories with in-hospital mortality.

We assessed the discriminative performance of each disease severity score in younger and older patients using a receiver operator characteristic (ROC) curve with area under the curve (AUC) analysis and in-hospital mortality as outcome. We calculated the sensitivities, specificities, negative predictive values (NPV), and positive predictive values (PPV) using the optimal cut-off points of each ROC curve. This cut-off point was determined by the maximum sum of the sensitivity and specificity in the ROC curve. To appropriately evaluate the qSOFA score, the cut-off point as originally proposed by Seymour et al. (≥2) has also been included in the analysis [13].

The AUC, sensitivity, specificity, PPV and NPV were reported as mean (95%-confidence interval (CI)). We considered AUCs to be poor at 0.6 to 0.7, adequate at 0.7 to 0.8, good at 0.8 to 0.9, and excellent at 0.9 or higher [21]. Differences in AUC were considered to be significant if the mean of older patients was not included in the 95%-CI of the younger patients.

All data were analyzed using SPSS software (SPSS 23.0, IBM, New York, USA).

#### Sensitivity analyses

Differences between the AUCs between older and younger patients could be caused by the age per se or by differences in disease severity because we expected disease severity in older patients to be worse compared to younger patients. To investigate whether age or disease severity was responsible for the AUCs in older patients, we did two sensitivity analyses: First, we excluded patients with acute onset organ failure [22] in the older patients and compared the AUCs of the five most common disease severity scores with the AUCs including all older patients. Secondly, we excluded older patients with a DNR status from the group with older patients and compared the AUCs in this selection with the AUCs of all older patients, because we have previously shown that a DNR status is another predictor of mortality and consequently a sign of higher disease severity [18].

In a third sensitivity analysis, we assessed the impact on the AUCs of inclusion of the ED patients with non-urgent triage categories.

Finally, we performed sensitivity analyses to assess the impact of missing variables (with multiple imputation), type of hospital (urban or academic) and time of inclusion (first or second half of inclusion period) on the AUCs of older and younger ED patients.

## Results

**Fig. 1:**
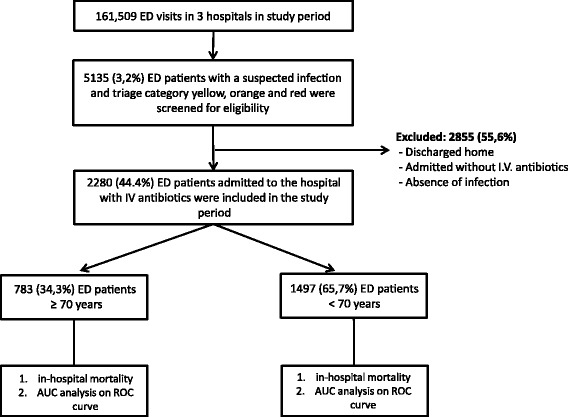
Patient flow through study

Figure 1 shows patient flow and inclusion criteria. A total of 2280 patients met the inclusion criteria and were enrolled in this study. Baseline characteristics are shown in Table 1. We included 1497 younger patients and 783 older patients. As expected, older patients had more comorbidities than younger patients and more often a DNR status. In line with the atypical symptom presentation in older patients, compared to younger patients, blood pressure was higher in older patients, while heart rate was lower in older patients. There were more patients with altered mental status in older patients.

**Table 1:** Patient characteristics of patients <70 and ≥70 years of age

	Total cohort	<70 years	≥70 years
Demographics			
N (%)	2280	1497 (65.7)	783 (34.3)
Age, mean (SD) [1]	61.1 (17.0)	52.2 (13.6)	78.2 (6.2)
Gender (male), n (%) [1]	1315 (57.7)	821 (54.8)	494 (63.1)
Included at University Medical Centre, n (%)	1860 (81.6)	1271 (84.9)	589 (75.2)
Co-morbidities, n (%)			
COPD [2]	349 (15.3)	158 (10.6)	191 (24.4)
Heart failure [1]	329 (14.4)	133 (8.9)	196 (25.0)
Liver disease [1]	113 (5.0)	94 (6.3)	19 (2.4)
Renal disease [1]	416 (18.2)	261 (17.4)	155 (19.8)
Nursing home [2]	138 (6.1)	46 (3.1)	92 (11.7)
Immune-compromised [2]	951 (41.7)	720 (48.1)	231 (29.5)
Malignancy – [1]	253 (11.1)	155 (10.4)	98 (12.5)
Malignancy + [3]	345 (15.1)	244 (16.3)	101 (12.9)
DNR status (%) (6)	478 (21.0)	185 (12.4)	293 (37.4)
Suspected source of infection, n (%)			
Pulmonary	1059 (46.4)	620 (41.4)	439 (56.1)
Urogenital	675 (29.6)	397 (26.5)	278 (35.5)
Abdominal	396 (17.4)	273 (18.2)	123 (15.7)
Skin	198 (8.7)	145 (9.7)	53 (6.8)
Neurological	48 (2.1)	35 (2.3)	13 (1.7)
other	396 (17.4)	301 (20.1)	95 (12.1)
Vital signs on admission			
Systolic blood pressure, mean (SD) (241)	131.9 (25.9)	129.8 (23.5)	135.7 (29.3)
Heart rate, mean (SD) (45)	108.0 (20.3)	110.2 (19.7)	103.8 (20.9)
Respiratory rate, mean (SD) (558)	24.0 (7.2)	22.83 (6.9)	25.73 (7.5)
Oxygen saturation, mean (SD) (71)	95.2 (4.8)	95.8 (4.6)	94.1 (5.2)
Temperature (°C), mean (SD) (94)	38.72 (1.11)	38.74 (1.06)	38.69 (1.20)
Altered mental status n (%) (420)	370 (16.2)	161 (10.8)	209 (26.7)
Laboratory analysis on admission			
Lactate (mmol/l), median (IQR) (293)	1.9 (1.4–2.6)	1.8 (1.4–2.6)	2.0 (1.5–2.8)
Platelets (×10^9^/l), median (IQR) (41)	209 (151–280)	207 (148–279)	213.5 (156–286)
INR median (IQR), (702)	1.1 (1–1.4)	1.1 (1.0–1.29)	1.2 (1.0–2.4)
Creatinine (μg/l), median (IQR) [16]	87 (67–120)	83 (64–110)	95 (74–134)
Urea (mmol/l), median (IQR) (40)	7.0 (5.1–10.2)	6.2 (4.6–8.8)	8.8 (6.6–12.5)
Band cells >5%, n (%)	179 (7.9)	103 (6.9)	76 (9.7)
Bilirubin (μmol/l), median (IQR) (349)	12 (8–19)	12 (8–18)	12 (9–20)
Treatment variables			
Time to antibiotics (min), median (IQR) [23]	103 (58–167)	109 (63–174)	91 (51–152)
Fluid resuscitation (L during ED stay), median (IQR)	1 (0.5–1.5)	1 (0.5–1.5)	1 (0.5–1.5)
Supplementary oxygen (L/min), median (IQR) (85)	3 (0–5)	2 (0–5)	3 (2–6)

Missing values are shown in parentheses for every variable

Abbreviations: *COPD* chronic obstructive pulmonary disease, *DNR* do not resuscitate, *°C* degrees Celsius, *SD* standard deviation, *IQR* interquartile range, *L* litre, min = minute

Table 2 shows the individual disease severity scores upon ED arrival. On average, older patients accumulate more points than younger patients. This trend persists not only in scores that include age and predisposition factors, i.e. PIRO and MEDS, but also in scores that consists only of acute physiology variables, i.e. qSOFA, MEWS and NEWS. Our primary outcome in-hospital mortality was significantly higher in older patients compared to younger patients, 9.5% (7.4–11.6) versus 4.6% (3.5–5.7), respectively, as shown in Table 3.

**Table 2:** Disease severity scores of patients <70 and ≥70 years of age

	Total cohort	<70 years	≥70 years
Disease severity scores			
MEDS, median (IQR)	5 (3–8)	5 (2–6)	8 (6–11)
PIRO, median (IQR)	10 (5–14)	8 (4–12)	13 (9–16)
qSOFA, median (IQR)	1 (0–1)	0 (0–1)	1 (0–1)
Acute onset organ failure^a^, median (IQR) [1] [n (%)]	0 (0–1) 592 (26)	0 (0–0) 344 (23.0)	0 (0–1) 248 (31.7)
MEWS, median (IQR)	5 (3–6)	4 (3–6)	5 (4–7)
NEWS, median (IQR)	6 (4–9)	6 (3–8)	8 (5–10)

^a^= According to Dellinger [22]. Missing values are shown in parentheses for every variable

*IQR* interquartile range, *qSOFA* quick sepsis-related organ dysfunction assessment score, *PIRO* predisposition, infection, response, and organ dysfunction score, *MEDS* mortality in emergency department sepsis score, *MEWS* modified early warning score, *NEWS* national early warning score

**Table 3:** Outcomes of patients <70 and ≥70 years of age

	Total cohort	<70 years	≥70 years
In-hospital mortality, N, % (95% CI) (90)	143, 6.3 (5.3–7.3)	69, 4.6 (3.5–5.7)	74, 9.5 (7.4–11.6)
ICU/MCU admission, n (%)^a^	220, 9.6 (8.4–10.8)	135, 9.0 (7.6–10.4)	85, 10.9 (8.7–13.1)
Unanticipated transfer, n (%)^b^ (101)	91, 4.0 (3.2–4.8)	63, 4.2 (3.2–5.2))	28, 3.6 (2.3–4.9)
Composite outcome, n (%)^c^	384, 16.8 (15.3–18.3)	225, 15 (13.2–16.8)	159, 20.3 (17.5–23.1)

Outcomes are reported as number (N), % (95%-CI) Missing values are shown in parentheses for every variable

^a^direct transfer from ED to MCU/ICU

^b^patient had been admitted from ED to ward, but had an unanticipated transfer to ICU or MCU within 48 h after admission

^c^combined outcome of ICU/MCU admission, unanticipated transfer and mortality

Abbreviations: *CI* Confidence interval, *ICU* Intensive care unit, *MCU* Medium care unit, *ED* Emergency department

**Fig. 2:**
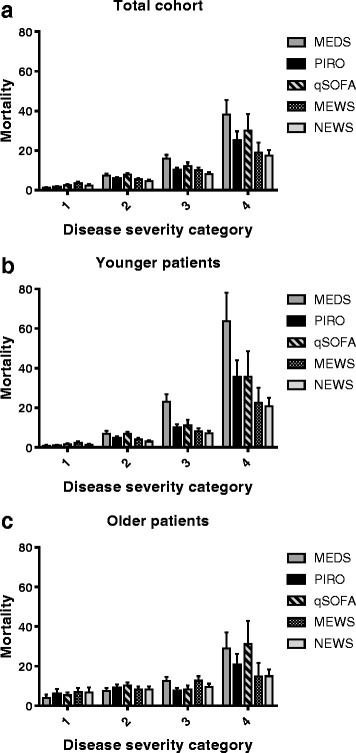
Prognostic performance of five disease severity scores. In-hospital mortality (%) as a function of disease severity categories of all disease severity scores in the total cohort (**a**), patients <70 years (**b**), and patients >70 years (**c**) was shown. Each disease severity score was divided into 4 categories to allow comparison among the 5 individual scores: low (PIRO 0–6, qSOFA 0, MEDS 0–5, MEWS 0–3 and NEWS 0–3), moderate (PIRO 7–12, qSOFA 1, MEDS 6–9, MEWS 4–6 and NEWS 4–7), high (PIRO 13–18, qSOFA 2, MEDS 10–15, MEWS 7–9 and NEWS 8–11) and severe (PIRO ≥19, qSOFA 3, MEDS ≥16, MEWS ≥10 and NEWS ≥12). These values were chosen taking into account the individual score guidelines to best represent comparable disease severity categories

We assessed the prognostic performance of each disease severity score by calculating the frequency of in-hospital mortality for each disease severity category, as shown in Fig. 2. Figure 2a shows that in the total cohort, there is a gradual increase of in-hospital mortality with an increase of disease severity category. This association is more pronounced in younger patients, resulting in a stronger increase in mortality with increasing disease severity compared to the total cohort. The association between disease severity and in-hospital mortality is almost absent in older patients.

**Fig. 3:**
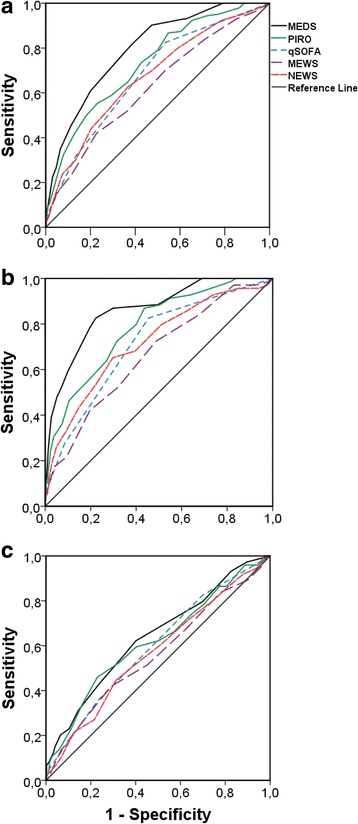
Discriminative performance of five disease severity scores in the total cohort (**a**), patients <70 years (**b**), and patients >70 years (**c**)

In addition to the prognostic performance of the disease severity scores, we also assessed the discriminative performance of each disease severity score. Figure 3a shows the ROC curves of the total cohort, revealing a large variation among the AUCs of the five scores. In the total cohort, the MEDS performed best with an AUC of 0.80 (0.76–0.83), whereas the MEWS performed worst with an AUC of 0.63 (0.58–0.67). The AUCs of all disease severity scores were larger in younger patients, as shown in Fig. 3b. In younger patients, the MEDS also performed best with an AUC of 0.86 (0.82–0.91), whereas the MEWS performed worst with an AUC of 0.66 (0.60–0.73). Corresponding to the younger patients, MEDS performed best in older patients although the AUC of 0.64 (0.57–0.71) was significantly lower than the AUC in younger patients. In older patients the MEWS also performed worst, with an AUC of 0.56 (0.49–0.63).

Table 4 shows the sensitivity, specificity, PPV and NPV using the optimal cutoff values of the ROC curves. Both the optimal qSOFA cutoff point that we found (≥1) and the cutoff value according to Seymour et al. (≥2) are shown.

**Table 4:** Discriminative performance of individual disease severity scores for each group

	AUC	Sensitivity	Specificity	PPV	NPV
Total cohort					
MEDS (≥7)	0.80 (0.76–0.83)	0.81 (0.80–0.83)	0.62 (0.60–0.64)	0.12 (0.10–0.13)	0.98 (0.97–0.99)
PIRO (≥14)	0.73 (0.69–0.77)	0.55 (0.53–0.57)	0.77 (0.75–0.79)	0.12 (0.11–0.14)	0.96 (0.95–0.97)
qSOFA (≥1)	0.68 (0.63–0.72)	0.83 (0.81–0.84)	0.47 (0.44–0.49)	0.10 (0.09–0.11)	0.98 (0.97–0.98)
qSOFA (≥2)	0.68 (0.63–0.72)	0.32 (0.30–0.33)	0.87 (0.85–0.88)	0.11 (0.10–0.12)	0.95 (0.94–0.96)
MEWS (≥7)	0.63 (0.58–0.67)	0.42 (0.40–0.44)	0.77 (0.76–0.79)	0.11 (0.10–0.13)	0.95 (0.94–0.96)
NEWS (≥8)	0.67 (0.62–0.72)	0.63 (0.61–0.65)	0.63 (0.61–0.65)	0.10 (0.08–0.11)	0.96 (0.95–0.97)
Patients age < 70					
MEDS (≥7)	0.86 (0.82–0.91)	0.83 (0.81–0.85)	0.78 (0.76–0.80)	0.14 (0.12–0.15)	0.99 (0.98–0.99)
PIRO (≥9)	0.78 (0.73–0.84)	0.87 (0.85–0.89)	0.56 (0.54–0.59)	0.08 (0.07–0.10)	0.99 (0.98–0.99)
qSOFA (≥1)	0.72 (0.66–0.78)	0.83 (0.81–0.85)	0.55 (0.52–0.57)	0.08 (0.06–0.09)	0.98 (0.98–0.99)
qSOFA (≥2)	0.72 (0.66–0.78)	0.28 (0.25–0.30)	0.92 (0.90–0.93)	0.10 (0.08–0.11)	0.96 (0.95–0.97)
MEWS (≥5)	0.66 (0.60–0.73)	0.73 (0.70–0.75)	0.51 (0.49–0.54)	0.06 (0.05–0.08)	0.97 (0.97–0.98)
NEWS (≥8)	0.72 (0.65–0.78)	0.65 (0.63–0.68)	0.70 (0.68–0.73)	0.09 (0.07–0.10)	0.98 (0.97–0.98)
Patients age > =70					
MEDS (≥9)	0.64 (0.57–0.71)	0.62 (0.59–0.66)	0.60 (0.56–0.63)	0.13 (0.11–0.16)	0.94 (0.92–0.95)
PIRO (≥16)	0.62 (0.55–0.69)	0.46 (0.42–0.49)	0.77 (0.74–0.80)	0.15 (0.13–0.18)	0.93 (0.91–0.95)
qSOFA (≥1)	0.60 (0.53–0.66)	0.82 (0.80–0.85)	0.30 (0.27–0.33)	0.11 (0.09–0.13)	0.94 (0.92–0.95)
qSOFA (≥2)	0.60 (0.53–0.66)	0.35 (0.32–0.38)	0.77 (0.74–0.80)	0.12 (0.10–0.14)	0.92 (0.90–0.94)
MEWS (≥7)	0.56 (0.49–0.63)	0.42 (0.38–0.45)	0.71 (0.68–0.74)	0.12 (0.10–0.14)	0.92 (0.90–0.94)
NEWS (≥10)	0.57 (0.50–0.64)	0.45 (0.41–0.48)	0.69 (0.66–0.72)	0.12 (0.10–0.14)	0.92 (0.90–0.94)

Optimal cut-off points are shown in parentheses for every disease severity score. Outcomes are shown as mean with 95% confidence interval

Abbreviations: *qSOFA* quick sepsis-related organ dysfunction assessment score, *PIRO* predisposition, infection, response, and organ dysfunction score, *MEDS* mortality in emergency department sepsis score, *MEWS* modified early warning score, *NEWS* national early warning score, *AUC* Area under the curve, *PPV* Positive predictive value, *NPV* Negative predictive value

In the total cohort, qSOFA (≥1) had the best sensitivity: 0.83 (0.81–0.84), whereas qSOFA (≥2) had the best specificity: 0.87 (0.85–0.88). In younger patients PIRO had the highest sensitivity: 0.87 (0.85–0.89); whereas qSOFA (≥2) had the highest specificity: 0.92 (0.90–0.93). In older patients the qSOFA (≥1) had the highest sensitivity: 0.82 (0.80–0.85); whereas the PIRO and qSOFA (≥2) had the highest specificity 0.77 (0.74–0.80). In younger patients, MEDS and PIRO approach a perfect NPV: 0.99 (0.98–0.99).

As shown in Additional file 3, the NEWS performed best at predicting ICU and MCU admission in the total cohort and younger patients, with AUCs of 0.75 (0.72–0.79) and 0.80 (0.76–0.84), respectively. In older patients the MEWS performed best with an AUC of 0.67 (0.61–0.73). MEDS and PIRO scores performed best at predicting the composite outcome in the total cohort, younger and older patients: 0.72 (0.70–0.75), 0.78 (0.74–0.81) and 0.61 (0.56–0.66), respectively.

The sensitivity analysis in Additional file 4 shows that exclusion of older patients with acute organ dysfunction from the older group decreases the AUCs of all disease severity scores from 0.56–0.64 to 0.43–0.59. Excluding patients with a DNR status did not affect the discriminative performance. Finally, 91 ED patients had non-urgent triage categories but had been admitted with intravenous antibiotics. Twenty-nine (32%) of the 91 patients were older 70 years. In-hospital mortality of the older patients was 45%, higher than the 8% (*P* < 0.001) of the younger patients who had been admitted with a non-urgent triage category. Inclusion of the 91 ED patients with non-urgent triage categories did not affect the AUCs of Fig. 3. In Additional file 5, the sensitivity analyses to assess the impact of missing variables, type of hospital and time of inclusion showed that the AUCs of older patients were structurally lower than in younger patients.

In summary, all scores performed structurally worse in older compared to younger patients. Scores which take comorbidities into consideration (i.e. MEDS and PIRO) consistently outperform disease severity scores that are solely based on acute physiology variables (i.e. qSOFA, NEWS and MEWS).

## Discussion

The main conclusion of the present study is that the most commonly used disease severity scores are less useful for risk stratification of older ED sepsis patients.

To the best of our knowledge, this is the first study directly comparing the prognostic and discriminative performance of the most commonly used disease severity scores in older and younger ED sepsis patients. In contrast to the findings of our study in ED sepsis patients, previous studies investigating the prognostic and discriminative performance of the MEWS in all older hospitalized patients concluded that the score is appropriate for risk stratification of in-hospital mortality. Cei et al. found a gradual increase of mortality in all disease severity categories, and Dundar et al. found an AUC of 0.89 [23, 24]. The discrepancy with our results is probably explained by different inclusion criteria resulting in different study populations: First, in these studies, patients older than 65 years of age were included. Secondly, in these studies not only older sepsis patients were included but older patients with all diagnoses. Most importantly, Dundar et al. included also patients who were discharged home after their ED presentation leading to a larger range of disease severity directly explaining the higher AUC in this study. Churpek et al. compared the MEWS in all older and younger patients who were admitted to a ward and concluded that it was inaccurate for prediction of the risk for cardiac arrest in older patients [14]. Studies in older trauma patients have shown that physiological parameters of older patients should be interpreted differently than those of younger patients. In these studies it was hypothesized that for example a systolic blood pressure of 90 mmHg is inappropriate for tissue perfusion of older people, therefore leading to acute organ failure [25–27]. We hypothesize that the same may be true for older sepsis patients, explaining the worse performance of all disease severity scores in older compared to younger patients tested in the present study. We used a cut-off of 70 years, as this is the cut-off which is also used in all Dutch government instated interventions for older people and 70 years is the official threshold to define older patients in the Netherland and is also the age above which vital signs start to deviate from younger patients. Nevertheless, in the future it may be necessary to develop scores for more than two age groups, comparable to the different reference values for vital signs in the paediatric patient population. These hypotheses are confirmed by Smith et al. who showed that there are different target values of vital signs for each age group of hospitalized ED patients [28].

Our study has another important finding. Because of the lack of a golden standard in sepsis research, there is a sepsis definition conference approximately every 15 years, aiming to improve our understanding of sepsis and better define it. The PIRO score was the newly proposed classification in the 2001 sepsis definition conference, and had a better prognostic and discriminative performance than the systemic inflammatory response syndrome (SIRS) definition proposed by Roger Bone in 1991, which it replaced [5–8]. The advantage of PIRO over the unidimensional SIRS is that PIRO distinguishes the unmodifiable predisposition and infection variables from the acute physiology variables, enabling better decision making in clinical practice. Recent studies showed that qSOFA outperforms SIRS in patients with a suspected infection outside the ICU, but it is unclear whether it is better than the PIRO score [29–32]. Our study shows that qSOFA has a worse prognostic and discriminative performance than MEDS and PIRO in older as well as in younger patients who were hospitalized with a suspected infection, possibly because PIRO and MEDS take age and comorbidities into consideration, whereas the qSOFA (as well as the NEWS and MEWS) are solely based upon acute physiology parameters.

Interestingly, we found that the rather old MEDS score still has the best prognostic and discriminative performance compared to all the newly developed scores. Instead of continuously developing new disease severity scores we suggest that it is more important to test the various scores in a randomized controlled trial to assess clinical acceptance and applicability, and impact on relevant clinical outcomes. After all, disease severity scores should aid in decision making in clinical practise and improve outcome of individual patients. This would also be in line with the GRADE guidelines and methodology [33].

The findings of the present study have several implications. First, recognition and risk stratification of older ED sepsis patients cannot be guided by the currently existing disease severity scores, as is indicated by the low AUCs in older ED patients. This is reinforced by the observation that all the disease severity scores only associate with in-hospital in the highest disease severity category, whereas in this range clinical judgement is often sufficient [8]. Secondly, a new prediction model specifically for older patients should be developed, similar to the separate scoring systems used in paediatric patients. Finally, we believe it would be premature to replace the PIRO classification by qSOFA.

Although our study has its strengths such as the large unselected sample size, incorporation of both tertiary and urban care centres, and inclusion of comorbidities to assess the performance of MEDS and PIRO, there are several limitations.

First, an observational study is subject to errors of documentation and data entry although we believe the prospective screening and inclusion of all consecutive patients in our database which is still done daily in the participating hospitals, minimizes this issue. Secondly, because older patients had a higher disease severity with more acute organ failure, it is possible that the treating physician was triggered to treat older patients more aggressively than younger patients leading to lower AUCs in younger patients, as have been argued previously [8]. However, both groups received similar treatment in terms of time to antibiotics, fluid resuscitation and supplemental oxygen. More importantly, the sensitivity analyses excluding the sicker patients from the older group showed comparable AUCs with the unselected older group, strongly suggesting that the lower discriminative performance in older patients is explained by age and not by the observation that older presented to the ED more ill. Secondly, our database included altered mental status as a binominal variable which could have led to difficulties calculating the MEWS score, which follows the AVPU-system. In a sample of 200 patients with altered mental status we determined that the grand majority had a Glasgow Coma Scale equal or higher than 14, thus the assumption to assign one point for altered mental status in the MEWS score seems realistic. In our study, urine production was not registered in our database. We therefore had to approach this variable by categorizing the serum creatinine levels in correspondence with the Dellinger definitions of renal organ failure (64–104 = 0, 105–141 = 1, 142–177 = 2, ≥178 = 3) [22]. We assumed that this sufficiently approached the urine production values. Nevertheless small deviations from the original MEWS may be present.

## Conclusions

We conclude that the most commonly used disease severity scores are less useful for risk stratification of older ED patients at risk for sepsis. Furthermore, our results question the replacement of PIRO by qSOFA. Future studies should invest in the development of a risk stratification tools specifically for older patients. In addition, a randomized controlled trial should establish which risk stratification tool is best in terms of impact on relevant clinical outcomes, and clinical applicability and acceptance.

## Additional files

Additional file 1: The quality improvement program with the standard screening procedure facilitating sepsis recognition, early ED resuscitation and disposition to an adequate level of care. (DOC 81 kb)

Additional file 2: Flow diagram used to assess the appropriateness of antibiotics. (PPT 136 kb)

Additional file 3: Discriminative performance with area under the curves (AUC) of secondary outcome measures. (DOCX 15 kb)

Additional file 4: Sensitivity analyses showing that exclusion of older patients with acute organ dysfunction or “Do Not Resuscitate (DNR) status” from the older group resulted in similar area under the curves (AUCs) of all disease severity scores. (DOCX 13 kb)

Additional file 5: Sensitivity analyses for assessment of the impact of missing data, type of hospital and period of inclusion on the discriminative performance of the disease severity score in older and younger patients. (DOCX 41 kb)

## Abbreviations

AUC: Area Under the Curve
AVPU: Alert Verbal Pain Unresponsive
CI: Confidence interval
COPD: Chronic Obstructive Pulmonary Disease
DNR: Do Not Resuscitate
ED: Emergency department
H: Hour
ICU: Intensive care unit
IQR: Inter Quartile Range
L: Liter
MCU: Medium Care unit
MEDS: Mortality in Emergency Department Sepsis
MEWS: Modified Early Warning Score
Min: Minute
NEWS: National Early Warning Score
PIRO: Predisposition, Infection, Response, Organ failure
qSOFA: Quick Sepsis-related Organ Failure Assessment
ROC: Receiver Operator Characteristics
SD: Standard Deviation
SEM: Standard Error of the Mean
