# Supplementary material for: The most commonly used disease severity scores are inappropriate for risk stratification of older emergency department sepsis patients: an observational multi-centre study
Source: Scand J Trauma Resusc Emerg Med. 2017 Sep 11;25:91. doi: 10.1186/s13049-017-0436-3 (PMC5594503; doi:10.1186/s13049-017-0436-3)
Supplement: Supplementary file 2 — Flow diagram used to assess the appropriateness of antibiotics. (PPT 136 kb) [file 13049_2017_436_MOESM2_ESM.ppt]

## Slide 1
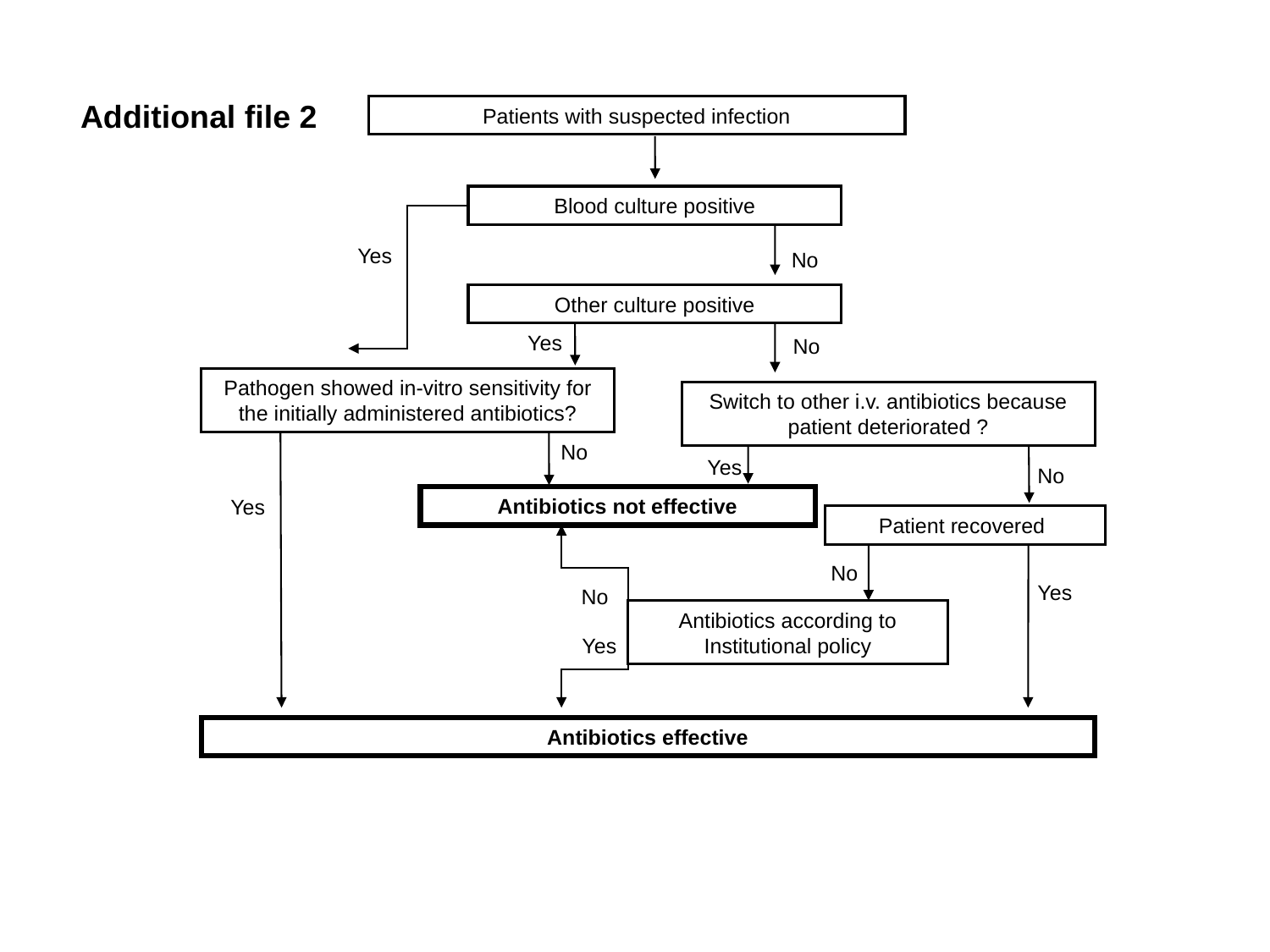

Additional file 2
Patients with suspected infection
Blood culture positive
Yes
No
Other culture positive
Yes
No
Pathogen showed in-vitro sensitivity for the initially administered antibiotics?
Switch to other i.v. antibiotics because patient deteriorated ?
No
Yes
No
Antibiotics not effective
Yes
Patient recovered
No
Yes
No
Antibiotics according to Institutional policy
Yes
Antibiotics effective
