# Supplementary material for: The most commonly used disease severity scores are inappropriate for risk stratification of older emergency department sepsis patients: an observational multi-centre study
Source: Scand J Trauma Resusc Emerg Med. 2017 Sep 11;25:91. doi: 10.1186/s13049-017-0436-3 (PMC5594503; doi:10.1186/s13049-017-0436-3)
Supplement: Supplementary file 3 — Discriminative performance with area under the curves (AUC) of secondary outcome measures. (DOCX 15 kb) [file 13049_2017_436_MOESM3_ESM.docx]

| **Additional file 3:** Discriminative performance of secondary outcome measures. | | | |
| --- | --- | --- | --- |
|  | **Total cohort** | **<70 years** | **≥70 years** |
| **ICU/MCU admission** |  |  |  |
| MEDS, AUC (95%-CI) | 0.70 (0.66-0.73) | 0.75 (0.71-0.79) | 0.61 (0.55-0.67) |
| PIRO, AUC (95%-CI) | 0.72 (0.69-0.75) | 0.77 (0.73-0.81) | 0.63 (0.57-0.69) |
| qSOFA, AUC (95%-CI) | 0.72 (0.68-0.75) | 0.75 (0.71-0.80) | 0.64 (0.58-0.70) |
| MEWS, AUC (95%-CI) | 0.71 (0.68-0.75) | 0.73 (0.68-0.78) | 0.67 (0.61-0.73) |
| NEWS, AUC (95%-CI) | 0.75 (0.72-0.79) | 0.80 (0.76-0.84) | 0.66 (0.60-0.72) |
| **Composite outcome** |  |  |  |
| MEDS, AUC (95%-CI) | 0.72 (0.70-0.75) | 0.78 (0.74-0.81) | 0.61 (0.56-0.66) |
| PIRO, AUC (95%-CI) | 0.72 (0.69-0.74) | 0.77 (0.74-0.80) | 0.61 (0.56-0.66) |
| qSOFA, AUC (95%-CI) | 0.69 (0.66-0.72) | 0.73 (0.70-0.77) | 0.60 (0.55-0.65) |
| MEWS, AUC (95%-CI) | 0.67 (0.64-0.70) | 0.70 (0.66-0.74) | 0.61 (0.56-0.66) |
| NEWS, AUC (95%-CI) | 0.71 (0.68-0.74) | 0.76 (0.73-0.80) | 0.61 (0.56-0.66) |
| Secondary outcome measures were ICU or MCU admission, an unanticipated transfer to an ICU or MCU within 48 hours after being admitted to a ward, and the composite outcome of in-hospital mortality, ICU or MCU admission, or unanticipated transfer to an ICU or MCU within 48 hours.  MEDS: Mortality in emergency department sepsis score. PIRO: Predisposition, infection, response and organ failure score. qSOFA: Quick sequential organ failure assessment score. MEWS: Modified early warning score. NEWS: National early warning score.  Abbrviations: ICU: Intensive care unit. MCU: Medium care unit.AUC: Area under the curve. AUCs are presented with 95%-confidence intervals (CI). | | | |
