# Supplementary material for: The most commonly used disease severity scores are inappropriate for risk stratification of older emergency department sepsis patients: an observational multi-centre study
Source: Scand J Trauma Resusc Emerg Med. 2017 Sep 11;25:91. doi: 10.1186/s13049-017-0436-3 (PMC5594503; doi:10.1186/s13049-017-0436-3)
Supplement: Supplementary file 4 — Sensitivity analyses showing that exclusion of older patients with acute organ dysfunction or “Do Not Resuscitate (DNR) status” from the older group resulted in similar area under the curves (AUCs) of all disease severity scores. (DOCX 13 kb) [file 13049_2017_436_MOESM4_ESM.docx]

| **Additional file 4:** AUCs from older patients sensitivity analysis. | | | |
| --- | --- | --- | --- |
|  | **≥70 years** | **≥70 years excluding acute organ failure** | **≥70 years**  **excluding DNR** |
| MEDS, AUC (95% CI) | 0.64 (0.57-0.71) | 0.59 (0.49-0.69) | 0.66 (0.55-0.77) |
| PIRO, AUC (95% CI) | 0.62 (0.55-0.69) | 0.54 (0.44-0.65) | 0.62 (0.50-0.74) |
| qSOFA, AUC (95% CI) | 0.60 (0.53-0.66) | 0.54 (0.43-0.65) | 0.61 (0.49-0.72) |
| MEWS, AUC (95% CI) | 0.56 (0.49-0.63) | 0.43 (0.33-0.52) | 0.56 (0.44-0.68) |
| NEWS, AUC (95% CI) | 0.57 (0.50-0.64) | 0.47 (0.38-0.57) | 0.59 (0.47-0.70) |
