# Supplementary material for: The most commonly used disease severity scores are inappropriate for risk stratification of older emergency department sepsis patients: an observational multi-centre study
Source: Scand J Trauma Resusc Emerg Med. 2017 Sep 11;25:91. doi: 10.1186/s13049-017-0436-3 (PMC5594503; doi:10.1186/s13049-017-0436-3)
Supplement: Supplementary file 5 — Sensitivity analyses for assessment of the impact of missing data, type of hospital and period of inclusion on the discriminative performance of the disease severity score in older and younger patients. (DOCX 41 kb) [file 13049_2017_436_MOESM5_ESM.docx]

**Additional file 5.**

Sensitivity analysis 1. To investigate the effect of the missing data, especially respiratory rate and altered mental status, on the receiver operator characteristics (ROC) with area under the curve (AUC) analyses. We imputed data with multiple imputations function in SPSS. In the tables below it can be seen that imputation yielded similar AUCs as in the original data in which normal values were used in case a value was missing.

Total cohort (N=2280).

| **Area Under the Curve^a,d^** | | | | | | |
| --- | --- | --- | --- | --- | --- | --- |
| Imputation Number | Test Result Variable(s) | Area | Std. Error^b^ | Asymptotic Sig.^c^ | Asymptotic 95% Confidence Interval | |
|  |  |  |  |  | Lower Bound | Upper Bound |
| Original data | Totale PIRO score voor behandeling | ,731 | ,021 | ,000 | ,689 | ,772 |
|  | Totale MEDS score voor behandeling | ,795 | ,018 | ,000 | ,760 | ,830 |
|  | qSOFA score 0-3 | ,677 | ,023 | ,000 | ,633 | ,721 |
| 1 | Totale PIRO score voor behandeling | ,728 | ,022 | ,000 | ,684 | ,772 |
|  | Totale MEDS score voor behandeling | ,789 | ,018 | ,000 | ,753 | ,825 |
|  | qSOFA score 0-3 | ,677 | ,023 | ,000 | ,633 | ,721 |
| a. For split file Imputation Number = Original data, the test result variable(s): Totale PIRO score voor behandeling, Totale MEDS score voor behandeling, qSOFA score 0-3 has at least one tie between the positive actual state group and the negative actual state group. Statistics may be biased. | | | | | | |
| b. Under the nonparametric assumption | | | | | | |
| c. Null hypothesis: true area = 0.5 | | | | | | |
| d. For split file Imputation Number = 1, the test result variable(s): Totale PIRO score voor behandeling, Totale MEDS score voor behandeling, qSOFA score 0-3 has at least one tie between the positive actual state group and the negative actual state group. Statistics may be biased. | | | | | | |

Patients <70 years (N=1497).

| **Area Under the Curve^a,d^** | | | | | | |
| --- | --- | --- | --- | --- | --- | --- |
| Imputation Number | Test Result Variable(s) | Area | Std. Error^b^ | Asymptotic Sig.^c^ | Asymptotic 95% Confidence Interval | |
|  |  |  |  |  | Lower Bound | Upper Bound |
| Original data | Totale PIRO score voor behandeling | ,784 | ,027 | ,000 | ,731 | ,837 |
|  | Totale MEDS score voor behandeling | ,861 | ,023 | ,000 | ,816 | ,905 |
|  | qSOFA score 0-3 | ,715 | ,031 | ,000 | ,655 | ,776 |
| 1 | Totale PIRO score voor behandeling | ,776 | ,029 | ,000 | ,719 | ,834 |
|  | Totale MEDS score voor behandeling | ,847 | ,025 | ,000 | ,798 | ,896 |
|  | qSOFA score 0-3 | ,715 | ,031 | ,000 | ,655 | ,776 |
| a. For split file Imputation Number = Original data, the test result variable(s): Totale PIRO score voor behandeling, Totale MEDS score voor behandeling, qSOFA score 0-3 has at least one tie between the positive actual state group and the negative actual state group. Statistics may be biased. | | | | | | |
| b. Under the nonparametric assumption | | | | | | |
| c. Null hypothesis: true area = 0.5 | | | | | | |
| d. For split file Imputation Number = 1, the test result variable(s): Totale PIRO score voor behandeling, Totale MEDS score voor behandeling, qSOFA score 0-3 has at least one tie between the positive actual state group and the negative actual state group. Statistics may be biased. | | | | | | |

Patients ≥70 years (N=793).

| **Area Under the Curve^a,d^** | | | | | | |
| --- | --- | --- | --- | --- | --- | --- |
| Imputation Number | Test Result Variable(s) | Area | Std. Error^b^ | Asymptotic Sig.^c^ | Asymptotic 95% Confidence Interval | |
|  |  |  |  |  | Lower Bound | Upper Bound |
| Original data | Totale PIRO score voor behandeling | ,617 | ,037 | ,001 | ,545 | ,689 |
|  | Totale MEDS score voor behandeling | ,638 | ,035 | ,000 | ,570 | ,707 |
|  | qSOFA score 0-3 | ,595 | ,035 | ,007 | ,527 | ,663 |
| 1 | Totale PIRO score voor behandeling | ,626 | ,037 | ,000 | ,553 | ,698 |
|  | Totale MEDS score voor behandeling | ,646 | ,035 | ,000 | ,577 | ,714 |
|  | qSOFA score 0-3 | ,595 | ,035 | ,007 | ,527 | ,663 |
| a. For split file Imputation Number = Original data, the test result variable(s): Totale PIRO score voor behandeling, Totale MEDS score voor behandeling, qSOFA score 0-3 has at least one tie between the positive actual state group and the negative actual state group. Statistics may be biased. | | | | | | |
| b. Under the nonparametric assumption | | | | | | |
| c. Null hypothesis: true area = 0.5 | | | | | | |
| d. For split file Imputation Number = 1, the test result variable(s): Totale PIRO score voor behandeling, Totale MEDS score voor behandeling, qSOFA score 0-3 has at least one tie between the positive actual state group and the negative actual state group. Statistics may be biased. | | | | | | |

In the sensitivity analyses below it was investigated if the type of hospital (Urban or academic medical centre) or time of inclusion (first half of inclusion or second half of inclusion period) had an effect on the receiver operator characteristics (ROC) with area under the curve analysis (AUC). In both hospital types and in both time periods, the AUCs were structurally lower in older compared to the younger patients, similar as in the total cohort. The main conclusion of the present study is therefore not explained by the type of hospital or the time of inclusion.

Sensitivity analysis 2. Impact of type of hospital on the AUCs of the 5 scores between older and younger ED patients.

Academic hospital.

| **Area Under the Curve^a,d^** | | | | | | |
| --- | --- | --- | --- | --- | --- | --- |
| LeeftijdCats | Test Result Variable(s) | Area | Std. Error^b^ | Asymptotic Sig.^c^ | Asymptotic 95% Confidence Interval | |
|  |  |  |  |  | Lower Bound | Upper Bound |
| ,00 | Totale PIRO score voor behandeling | ,799 | ,029 | ,000 | ,741 | ,856 |
|  | Totale MEDS score voor behandeling | ,866 | ,024 | ,000 | ,819 | ,913 |
|  | qSOFA score 0-3 | ,722 | ,033 | ,000 | ,657 | ,788 |
|  | MEWS in 4 categorieën | ,694 | ,034 | ,000 | ,627 | ,760 |
|  | NEWS in 4 categoriën | ,684 | ,033 | ,000 | ,618 | ,749 |
| 1,00 | Totale PIRO score voor behandeling | ,630 | ,044 | ,003 | ,543 | ,716 |
|  | Totale MEDS score voor behandeling | ,630 | ,042 | ,003 | ,547 | ,712 |
|  | qSOFA score 0-3 | ,605 | ,045 | ,017 | ,516 | ,694 |
|  | MEWS in 4 categorieën | ,555 | ,047 | ,210 | ,462 | ,647 |
|  | NEWS in 4 categoriën | ,546 | ,043 | ,297 | ,461 | ,630 |
| a. For split file LeeftijdCats = ,00, the test result variable(s): Totale PIRO score voor behandeling, Totale MEDS score voor behandeling, qSOFA score 0-3, MEWS in 4 categorieën, NEWS in 4 categoriën has at least one tie between the positive actual state group and the negative actual state group. Statistics may be biased. | | | | | | |
| b. Under the nonparametric assumption | | | | | | |
| c. Null hypothesis: true area = 0.5 | | | | | | |
| d. For split file LeeftijdCats = 1,00, the test result variable(s): Totale PIRO score voor behandeling, Totale MEDS score voor behandeling, qSOFA score 0-3, MEWS in 4 categorieën, NEWS in 4 categoriën has at least one tie between the positive actual state group and the negative actual state group. Statistics may be biased. | | | | | | |

Urban hospitals.

| **Area Under the Curve^a,d^** | | | | | | |
| --- | --- | --- | --- | --- | --- | --- |
| LeeftijdCats | Test Result Variable(s) | Area | Std. Error^b^ | Asymptotic Sig.^c^ | Asymptotic 95% Confidence Interval | |
|  |  |  |  |  | Lower Bound | Upper Bound |
| ,00 | Totale PIRO score voor behandeling | ,717 | ,070 | ,015 | ,580 | ,853 |
|  | Totale MEDS score voor behandeling | ,860 | ,064 | ,000 | ,736 | ,985 |
|  | qSOFA score 0-3 | ,706 | ,081 | ,021 | ,548 | ,864 |
|  | MEWS in 4 categorieën | ,532 | ,080 | ,723 | ,376 | ,687 |
|  | NEWS in 4 categoriën | ,562 | ,077 | ,484 | ,410 | ,714 |
| 1,00 | Totale PIRO score voor behandeling | ,583 | ,067 | ,168 | ,452 | ,714 |
|  | Totale MEDS score voor behandeling | ,655 | ,064 | ,010 | ,530 | ,781 |
|  | qSOFA score 0-3 | ,549 | ,054 | ,419 | ,442 | ,655 |
|  | MEWS in 4 categorieën | ,537 | ,062 | ,540 | ,415 | ,658 |
|  | NEWS in 4 categoriën | ,518 | ,059 | ,772 | ,402 | ,633 |
| a. For split file LeeftijdCats = ,00, the test result variable(s): Totale PIRO score voor behandeling, Totale MEDS score voor behandeling, qSOFA score 0-3, MEWS in 4 categorieën, NEWS in 4 categoriën has at least one tie between the positive actual state group and the negative actual state group. Statistics may be biased. | | | | | | |
| b. Under the nonparametric assumption | | | | | | |
| c. Null hypothesis: true area = 0.5 | | | | | | |
| d. For split file LeeftijdCats = 1,00, the test result variable(s): Totale PIRO score voor behandeling, Totale MEDS score voor behandeling, qSOFA score 0-3, MEWS in 4 categorieën, NEWS in 4 categoriën has at least one tie between the positive actual state group and the negative actual state group. Statistics may be biased. | | | | | | |

Sensitivity analysis 3. Impact of inclusion period on AUCs of the 5 scores between older and younger ED patients.

First half of inclusion period.

| **Area Under the Curve^a,d^** | | | | | | |
| --- | --- | --- | --- | --- | --- | --- |
| LeeftijdCats | Test Result Variable(s) | Area | Std. Error^b^ | Asymptotic Sig.^c^ | Asymptotic 95% Confidence Interval | |
|  |  |  |  |  | Lower Bound | Upper Bound |
| ,00 | Totale PIRO score voor behandeling | ,783 | ,038 | ,000 | ,709 | ,858 |
|  | Totale MEDS score voor behandeling | ,892 | ,027 | ,000 | ,840 | ,945 |
|  | qSOFA score 0-3 | ,684 | ,044 | ,000 | ,597 | ,771 |
|  | NEWS in 4 categoriën | ,624 | ,044 | ,010 | ,538 | ,709 |
|  | MEWS in 4 categorieën2 | ,589 | ,052 | ,065 | ,488 | ,690 |
| 1,00 | Totale PIRO score voor behandeling | ,554 | ,047 | ,240 | ,461 | ,647 |
|  | Totale MEDS score voor behandeling | ,615 | ,048 | ,012 | ,520 | ,709 |
|  | qSOFA score 0-3 | ,554 | ,044 | ,239 | ,466 | ,641 |
|  | NEWS in 4 categoriën | ,500 | ,045 | ,999 | ,411 | ,589 |
|  | MEWS in 4 categorieën2 | ,547 | ,044 | ,302 | ,462 | ,632 |
| a. For split file LeeftijdCats = ,00, the test result variable(s): Totale PIRO score voor behandeling, Totale MEDS score voor behandeling, qSOFA score 0-3, NEWS in 4 categoriën, MEWS in 4 categorieën2 has at least one tie between the positive actual state group and the negative actual state group. Statistics may be biased. | | | | | | |
| b. Under the nonparametric assumption | | | | | | |
| c. Null hypothesis: true area = 0.5 | | | | | | |
| d. For split file LeeftijdCats = 1,00, the test result variable(s): Totale PIRO score voor behandeling, Totale MEDS score voor behandeling, qSOFA score 0-3, NEWS in 4 categoriën, MEWS in 4 categorieën2 has at least one tie between the positive actual state group and the negative actual state group. Statistics may be biased. | | | | | | |

Second half of inclusion period.

| **Area Under the Curve^a,d^** | | | | | | |
| --- | --- | --- | --- | --- | --- | --- |
| LeeftijdCats | Test Result Variable(s) | Area | Std. Error^b^ | Asymptotic Sig.^c^ | Asymptotic 95% Confidence Interval | |
|  |  |  |  |  | Lower Bound | Upper Bound |
| ,00 | Totale PIRO score voor behandeling | ,787 | ,038 | ,000 | ,712 | ,861 |
|  | Totale MEDS score voor behandeling | ,822 | ,037 | ,000 | ,750 | ,894 |
|  | qSOFA score 0-3 | ,751 | ,042 | ,000 | ,669 | ,833 |
|  | MEWS in 4 categorieën | ,727 | ,041 | ,000 | ,647 | ,807 |
|  | NEWS in 4 categoriën | ,708 | ,040 | ,000 | ,629 | ,787 |
| 1,00 | Totale PIRO score voor behandeling | ,717 | ,053 | ,000 | ,612 | ,822 |
|  | Totale MEDS score voor behandeling | ,682 | ,048 | ,001 | ,587 | ,777 |
|  | qSOFA score 0-3 | ,666 | ,053 | ,003 | ,562 | ,770 |
|  | MEWS in 4 categorieën | ,597 | ,063 | ,087 | ,474 | ,720 |
|  | NEWS in 4 categoriën | ,601 | ,052 | ,075 | ,500 | ,703 |
| a. For split file LeeftijdCats = ,00, the test result variable(s): Totale PIRO score voor behandeling, Totale MEDS score voor behandeling, qSOFA score 0-3, MEWS in 4 categorieën, NEWS in 4 categoriën has at least one tie between the positive actual state group and the negative actual state group. Statistics may be biased. | | | | | | |
| b. Under the nonparametric assumption | | | | | | |
| c. Null hypothesis: true area = 0.5 | | | | | | |
| d. For split file LeeftijdCats = 1,00, the test result variable(s): Totale PIRO score voor behandeling, Totale MEDS score voor behandeling, qSOFA score 0-3, MEWS in 4 categorieën, NEWS in 4 categoriën has at least one tie between the positive actual state group and the negative actual state group. Statistics may be biased. | | | | | | |
